# The Predictive Role of Baseline ^18^F-FDG PET/CT Radiomics in Follicular Lymphoma on Watchful Waiting: A Preliminary Study

**DOI:** 10.3390/diagnostics15040432

**Published:** 2025-02-11

**Authors:** Daria Maccora, Michele Guerreri, Rosalia Malafronte, Francesco D’Alò, Stefan Hohaus, Marco De Summa, Vittoria Rufini, Roberto Gatta, Luca Boldrini, Lucia Leccisotti, Salvatore Annunziata

**Affiliations:** 1 Nuclear Medicine Unit, IRCCS Regina Elena National Cancer Institute, Via Elio Chianesi, 53, 00144 Rome, Italy; 2 Section of Nuclear Medicine, Department of Radiological Sciences and Haematology, Università Cattolica del Sacro Cuore, L.go A. Gemelli, 8, 00168 Rome, Italy; 3 Department of Clinical and Experimental Sciences, University of Brescia, 25123 Brescia, Italy; 4 Hematology Unit, Fondazione Policlinico Universitario A. Gemelli IRCCS, 00168 Rome, Italy; 5 Section of Haematology, Department of Radiological Sciences and Haematology, Università Cattolica del Sacro Cuore, 00168 Rome, Italy; 6 Medipass S.p.a. Integrative Service, 00168 Rome, Italy; 7 Nuclear Medicine Unit, Fondazione Policlinico Universitario A. Gemelli IRCCS, L.go A. Gemelli, 8, 00168 Rome, Italy; 8 Radiotherapy Unit, Fondazione Policlinico Universitario A. Gemelli IRCCS, 00168 Rome, Italy

**Keywords:** follicular lymphoma, PET, FDG, metabolic tumour volume, radiomics

## Abstract

**Background**: Patients with low tumour burden follicular lymphoma (FL) are managed with an initial watchful waiting (WW) approach. The way to better predict the time-to-treatment (TTT) is still under investigation for its possible clinical impact. This study explored whether radiomic features extracted from baseline ^18^F-FDG PET/CT could predict TTT in FL patients on WW. **Methods**: Thirty-eight patients on initial WW (grade 1–3a) were retrospectively included from 2010 to 2019. Eighty-one PET/CT morphological and first-level intensity radiomic features were extracted from the total metabolic tumour burden (TMTV), the lesion having the highest SUVmax and a reference volume-of-interest placed on the healthy liver. Models using linear regression (LR) and support vector machine (SVM) were constructed to assess the feasibility of using radiomic features to predict TTT. A leave-one-out cross-validation approach was used to assess the performance. **Results**: For LR models, we found a root-mean-squared error of 29.4, 28.6, 26.4 and 26.8 and an R^2^ of 0.03, 0.08, 0.21 and 0.20, respectively, incrementing the features from one to four. Accordingly, the best model included three features: the liver minimum SUV value, the liver SUV skewness and the sum of squared SUV values in the TMTV. For SVM models, accuracies of 0.79, 0.63, 0.76 and 0.68 and areas under the curve of 0.80, 0.72, 0.77 and 0.63 were found, respectively, incrementing the features from one to four. The best performing model used one feature, namely the median value of the lesion containing the SUVmax value. **Conclusions**: The baseline PET/CT radiomic approach has the potential to predict TTT in FL patients on WW. Integrating radiomics with clinical parameters could further aid in patient stratification.

## 1. Introduction

Follicular lymphoma (FL) represents the second most common non-Hodgkin lymphoma subtype and the most frequent indolent lymphoma in Western countries [1,2]. FL most commonly has an indolent behaviour and may record a spontaneous remission, although cases with a more aggressive course may occur [3]. A watchful waiting (WW) strategy is adopted in patients with low tumour burden, no bulky disease and no symptoms, starting chemotherapy after the onset of symptoms [4]. Approximately 25–40% of FL patients managed with WW develop progression of disease within 24 months. Clinical prognostic models currently used at staging, including the Follicular Lymphoma International Prognostic Index (FLIPI) and the more recently described FLIPI2 [5,6], only poorly identify patients at an elevated risk of progression during WW [7].

Recent guidelines recommend ^18^F-FDG Positron Emission Tomography/Computed Tomography (PET/CT) in clinical practice as a standard of care in FL for diagnosis, baseline staging, suspicion of transformation, best suitable re-biopsy site in the context of relapse and at the time of response assessment (gold standard) [8,9]. Several studies demonstrated that higher values of the SUVmax, such as the maximum standardized uptake value, the total metabolic tumour volume (TMTV) and the Deauville score at baseline PET/CT evaluation in FL may be associated with an inferior survival time [10,11,12,13].

In the era of precision medicine, there is a growing interest in patient risk stratification at diagnosis using biomarkers or other data, and the introduction of radiomics appears in line with this. Radiomics is a high-throughput approach that translates medical images into minable data by extracting several quantitative features describing the intensity, shape and heterogeneity of targeted lesions. Radiomic models could indirectly be an expression of tumour biology behaviour and could be considered an additional support, together with clinical and histological data, to help the clinicians in the diagnostic process [14]. However, their application in lymphoma is limited due to the difficulty in collecting large patient cohorts given the lower incidence compared to other cancers, and particularly the subgroup of low tumour burden FL.

Various semi-quantitative parameters obtained from baseline PET/CT scans have been analysed in FL patients to assess the risk of progressive disease. Particularly, the most active lesion and the TMTV have been often investigated as functional elements able to predict FL patients’ outcome at diagnosis, but with conflicting results [11,12,13,15,16,17,18,19,20]. Similarly, the evaluation of the healthy organ ^18^F-FDG (FDG) uptake (i.e., liver) as another prognostic marker was considered promising [21,22]. The following step should be the application of radiomics to the baseline PET/CT with similar prognostic purposes.

The aim of the present study was to investigate whether radiomic features extracted from baseline PET/CT could help to predict the treatment starting time in low tumour burden FL patients on WW as well as their prognostic role by integrating healthy organ uptake data. Radiomics features were extracted from the metabolic tumour burden and from the lesion with the highest SUVmax as well as from the healthy liver. These extracted features were used to build predictive models for the time to treatment during WW (TTT), by using two different analytical algorithms. The model performances were validated using an internal cross-validation approach, to ensure reproducibility and robustness.

## 2. Methods

### 2.1. Subjects

We performed a retrospective analysis on low tumour burden FL patients recruited at the Haematology Unit of Fondazione Policlinico Universitario Agostino Gemelli IRCCS between January 2010 and December 2019. Inclusion criteria were age ≥18 years, histologically confirmed FL (grade 1 to 3a in accordance with the World Health Organization Classification [15]), initial WW for low tumour burden disease according to *Groupe d’Etude des Lymphomes Folliculaires* (GELF) criteria [4], a baseline positive whole body FDG PET/CT and at least 24 months of follow-up. A positive FDG PET/CT referred to a scan with one or more focal uptake, corresponding to one or more lesions. Exclusion criteria were as follows: (1) patients with other malignant tumours, (2) known histological transformation at baseline, (3) aggressive B-cell lymphoma, (4) FL grade 3b according to the WHO classification and (5) no available PET/CT at baseline. Medical records were analysed to extract clinical information, such as baseline and follow-up data. The cohort of the present study is a subgroup of patients that had previously been analysed and published [15].

Thirty-eight patients were included in this study (19 male and 19 female), with a median age of 61 years (range 34–85). Twenty-five patients (66%) had an advanced disease stage (III or IV), while twenty-three patients (60%) an intermediate-high (≥2) FLIPI score. Table 1 shows other patient and disease characteristics. In this study population, 26 patients (68%) started immuno-chemotherapy due to disease progression after a median follow-up of 75 months (range 15–134).

TTT is the time between the initial diagnosis and the beginning of the systemic therapy. Median TTT in this study population resulted 40 months (range 3–134).

This retrospective study was conducted according to the institutional ethical guidelines and in accordance with the Declaration of Helsinki. The retrospective data collection and anonymous analysis was approved by our Institutional Review Board.

### 2.2. FDG PET/CT Imaging

PET/CT studies were acquired according to European Association of Nuclear Medicine guidelines [23]. Images were acquired using either a Gemini GXL (Philips Healthcare, Cleveland, OH, USA) or Biograph mCT (Siemens Healthineers, Erlangen, Germany) scanner, applying the respective standard reconstruction protocol. PET/CT studies were then transferred to a commercially available multimodality imaging application for oncology.

### 2.3. Image Segmentation

PET/CT exams were reviewed by two experienced nuclear physicians, blinded to patients’ clinical and follow-up data. A visual assessment and a semi-quantitative analysis were performed. A software package for a semiautomatic segmentation (PET_Edge_, version 7.0.5 MIM Encore Software Inc., Cleveland, OH) was used to obtain volumes of interest (VOIs). This contouring method has been validated in a multi-observer study that demonstrated its superiority over manual and threshold methods [24]. Specifically, a 2 cm–diameter default spherical VOI was manually positioned on the right lobe of the healthy liver, which served as a reference for the lesions’ identification. A PET Response Criteria in Solid Tumors (PERCIST)–based background threshold (liver) allowed to contour hypermetabolic lesions (nodal and extra nodal). It was calculated using the following formula: PERCIST = (1.5 × liver mean) + (2 × liver standard deviation) [16]. Physicians were required to reject false–positive lesions (sites of physiological FDG uptake, external contamination and pathologic uptake deemed lymphoma-unrelated) before all approved VOIs were computed to obtain TMTV. Bone marrow and spleen were included in the volume measurement only if there was focal uptake. Inter-observer reproducibility of PET measurements was analysed and was high (intraclass correlation coefficient = 0.92 with 95% confidence interval [CI] 0.89–0.95). Semi-quantitative parameters (SUVmax and SUVmean) and metabolic tumour volume (MTV) were extracted for each lesion. SUVmax and SUVmean are defined as the greatest uptake in a single voxel within the semi-automatically defined VOI and the average SUV throughout the VOI, respectively. MTV is defined as the volume of tumour tissues with increased FDG uptake above the described threshold method. The TMTV was calculated as the sum of the MTVs.

### 2.4. Radiomic Feature Extraction

The IBSI-compliant [25] MODDICOM software [26] (https://github.com/kbolab/moddicom, 1 November 2024) was used for the feature extraction from PET images. The extracted features included first-level statistical and morphological features (see Supplementary Materials). Due to the inherent presence of multiple distinct VOIs, placed on the different lesions, we opted for a diversified feature estimation approach for the two types of features: on one hand, the first-level statistical were computed over the entire tumour burden, considering all the voxels belonging to the different lesion VOIs simultaneously. On the other hand, the morphological features, which include information such as sphericity that cannot be computed over multiple separated regions, were first computed in each lesion separately and then their values were averaged. The number of features extracted was 31 (17/14 first-level statistical/morphological).

In addition to the features computed over the entire tumour burden, first-level statistical and morphological features computed in two specific regions were also included. The first region that we identified was the one including the overall SUVmax. The features extracted from this region were labelled with the suffix “SUVmax”. The second region that we considered was the reference VOI positioned on the healthy liver. The features computed in this VOI were labelled with the suffix “liver”. In this case, the morphological features were discarded. The choice of this VOI was motivated by previous studies, which found that the uptake of healthy organs (i.e., liver, cerebellum) could reflect the tumour burden and substitute MTV. Particularly, the hepatic uptake was reported to be inversely correlated to TMTV [21,22].

Finally, the total number of lesions for each patient as well as the TMTV expressed in cm^3^ were added as extra features. The total number of features extracted was *N* = 82.

### 2.5. Differences Between Scanners and Feature Harmonization

Two different PET scanners were used to acquire the examinations. Sixteen scans were acquired on scanner 1 and the remaining twenty-two on scanner 2. We assessed the presence of statistically significant differences between the features extracted from the two scanners, using a non-parametric Wilcoxon—Mann–Whitney test. A *p*-value of 0.05 after multiple comparison correction based on the false discovery rate (FDR) method was considered significant. The same statistical test was used to check whether there was a statistical difference between the TTT values of examinations acquired using different scanners.

To reduce the scanner-dependent differences found between the features, the ComBat harmonization method was implemented. ComBat was originally proposed in the field of genomics [27] and has ever since been largely used in the medical imaging field [28] including the radiomic field [29,30,31,32]. A single-effect term accounting for the scanner type (either Gemini GXL or Biograph mCT) was considered in the analysis.

### 2.6. Regression and Classification Model Definition

We selected two different models to investigate the potential of using the PET-derived radiomic features to estimate the TTT. Since TTT is a continuous variable, we implemented a linear regression (LR) model that outputs continuous values. In addition, we applied a support vector machine (SVM) model that was intended to classify whether, given a set of features, the TTT lies before or after the median value of the TTT distribution. The SVM model was executed with a radial kernel. Both models were implemented in R (version 4.3.0) using the package “tidyModels”. The metrics used to assess the model performance were the R^2^ and root-mean-squared error (RMSE) for the LR and the accuracy, precision and sensitivity as well as receiver operating characteristic (ROC) curves and the corresponding area under the curve (AUC) for the SVM model.

### 2.7. Feature Selection

Due to the small sample size and the large number of features, a model constructed using all the available features will most probably overfit the data. Hence, a feature selection step was implemented to determine which among the extracted features had the potential to best explain the TTT. Specifically, a forward feature selection approach tailored for each of the different models was applied. First, we built mono-variate models using each of the available features and looking for the feature that gave the best fit performance. The best feature was retained and used to iteratively construct a bi-variate model using the remaining N-1 features. The best feature pair was retained and used to construct more complex models. This approach was iterated up to four predictors. This feature selection approach was performed for both the LR and SVM models. The fit performance was assessed over the entire subject cohort, in terms of R^2^ for the LR model and in terms of accuracy for the SVM model. Prior to the fit, all the features were standardized using z-score to ensure equal contribution to the fit convergence.

In addition, we assessed whether there was an association between some of the clinical variables and the radiomic features extracted from the PET images and this can be found in the Supplementary Materials.

### 2.8. Model Selection

To assess how well the predictive models defined by the feature selection step generalize to an unseen data, a validation step was implemented. The model performances were validated using an internal cross-validation approach. Due to the relatively small sample size, which prevents to split the dataset into a training and validation set, we opted for a leave-one-out cross-validation (LOOCV) approach. The dataset was divided into K subsets, where K is the number of subjects in our cohort. The datasets were constructed by holding out one data point as the validation set, while the models were trained on the remaining K-1 data points. This process was repeated several times and each time excluding a different data point as validation. After K iterations, the relevant performance metrics defined above were computed on the K predictions from the test set. The same metrics were computed on the training set for comparison. In this case, K different metric values were computed for each iteration on the K-1 data used for training. The obtained values were then averaged to obtain unique training metrics for each model.

### 2.9. Kaplan–Meier Analysis

A Kaplan–Meier analysis was also applied to assess whether the radiomic features extracted from the PET images could be used to divide only the patients who started chemotherapy after the onset of progressive disease into groups characterized by significantly different times in which the TTT event occurs. The Kaplan–Meier analysis was implemented following these steps: first, we iterated over each of the features, computing the median value and dividing the patients in two groups, the patients with feature value higher than the median value and those with a lower feature value; next, a log-rank test was conducted examining how the TTT events in each group unfold sequentially in time. In order to control for multiple comparisons, the *p*-values obtained from the log-rank test were adjusted using the FDR method. Adjusted *p*-values smaller than 0.05 were considered statistically significant.

## 3. Results

### 3.1. Contoured Lesions and PET Measurement Reproducibility

**Figure 1:**
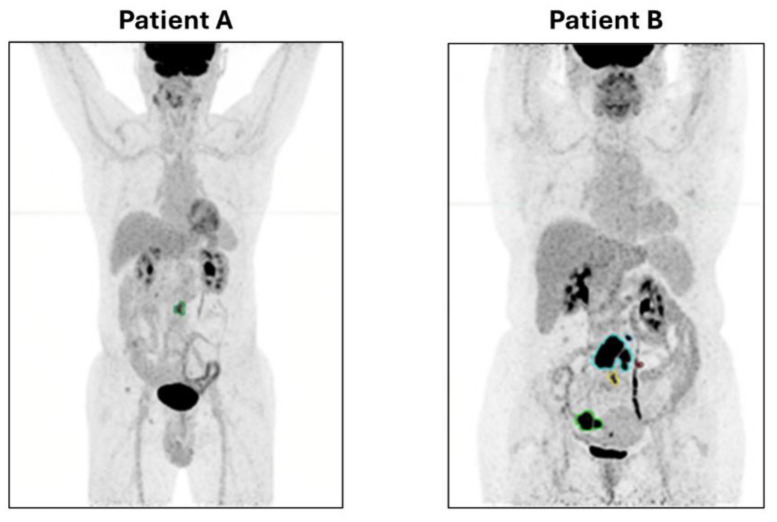
Total metabolic tumour volume (TMTV) delineated at baseline FDG PET/CT using an automatic whole-body segmentation software. On the left side (Patient A), a baseline PET/CT of a male patient with low tumour burden follicular lymphoma (FL). He is 72 years old, with a disease grading of 2, a stage IVA, a Follicular Lymphoma International Prognostic Index (FLIPI) of 3 and a TMTV of 5.0 cm^3^, on watch and wait at the last follow-up (54 months). On the right side (Patient B), a baseline PET/CT of a 69-year-old female patient with low tumour burden FL: grading 2, stage IIA, FLIPI = 1 and with a TMTV of 48.9 cm^3^, treated with R-BENDA (6 cycles) for disease progression 7 months after the initial diagnosis.

The median number of lesions contoured on PET/CT were 7 per-patient (range 2–13), and the median TMTV was 22.8 cm^3^, ranging from 6.2 to 52.0 cm^3^. Table 2 shows the baseline FDG PET/CT parameters. Two representative cases are reported in Figure 1.

### 3.2. Difference Between Scanners and ComBat-Based Feature Harmonization

A total of 56 out of 82 features were found significantly different between the groups of examinations acquired with different scanners. No significant difference was found between the TTT of the two groups.

**Figure 2:**
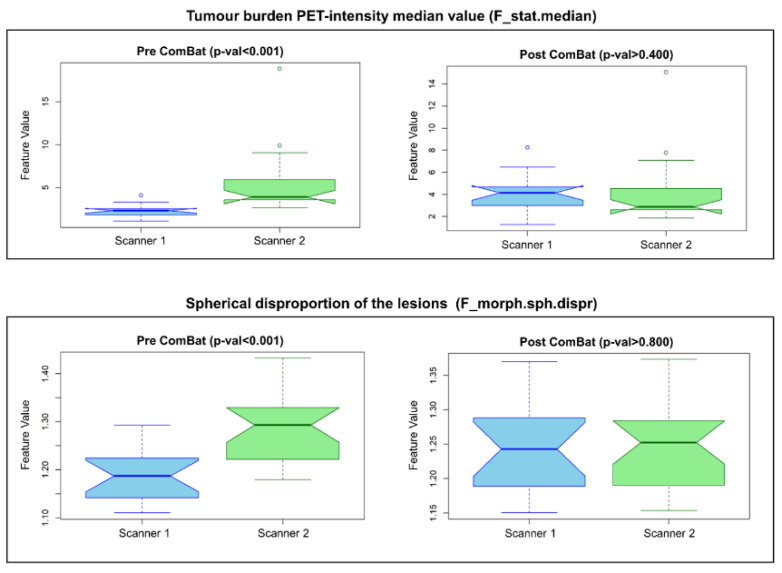
ComBat method application. We report two representative features extracted from the PET images that underwent the ComBat harmonization process to account for scanner difference. The boxplots of feature values computed for the patients acquired with different scanners before harmonization are reported on the left-hand side. The same features after ComBat harmonization are reported on the right. The median value of the PET intensity computed in the metabolic tumour burden (a first-level intensity feature) is reported on the top, while the spherical disproportion (a morphological feature) is reported on the bottom of the figure.

After ComBat application, the number of significantly different features decreased from 56 to 2. Two representative feature values grouped with respect to the scanners used for the acquisition, before and after the application of the ComBat harmonization technique, are reported in Figure 2. In both cases, a statistically significant difference was found between the groups, but the difference was reduced after ComBat. The features that remained statistically different after ComBat application were discarded from the analysis.

### 3.3. Feature Selection

A forward feature selection method was employed, and the features were presented in sequential order, starting from the first selected feature up to the one selected as the last.

The features that were found to be the most significant for the linear regression LR model were the minimum SUV value found in the liver (F_stat.min.liver), the skewness of the SUV values found in the liver (F_stat.skew.liver), the sum of squares of the intensity values in the entire tumour burden (F_stat.uniformity) and the morphological spherical disproportion found in the SUVmax VOI (F_morph.sph.dispr.SUVmax). The features found to be significant for the SVM model were the SUV median values computed in the SUVmax region (F_stat.median.SUVmax), the 10th percentile of the intensity distribution from the same region (F_stat.10thpercentile.SUVmax), the signal distribution kurtosis computed in the liver (F_stat.kurt.liver) as well as the kurtosis computed in the SUVmax region (F_stat.kurt.SUVmax).

The relevant features as well as the performance obtained for each of the different models are summarized in Table 3.

### 3.4. Model Performance

**Figure 3:**
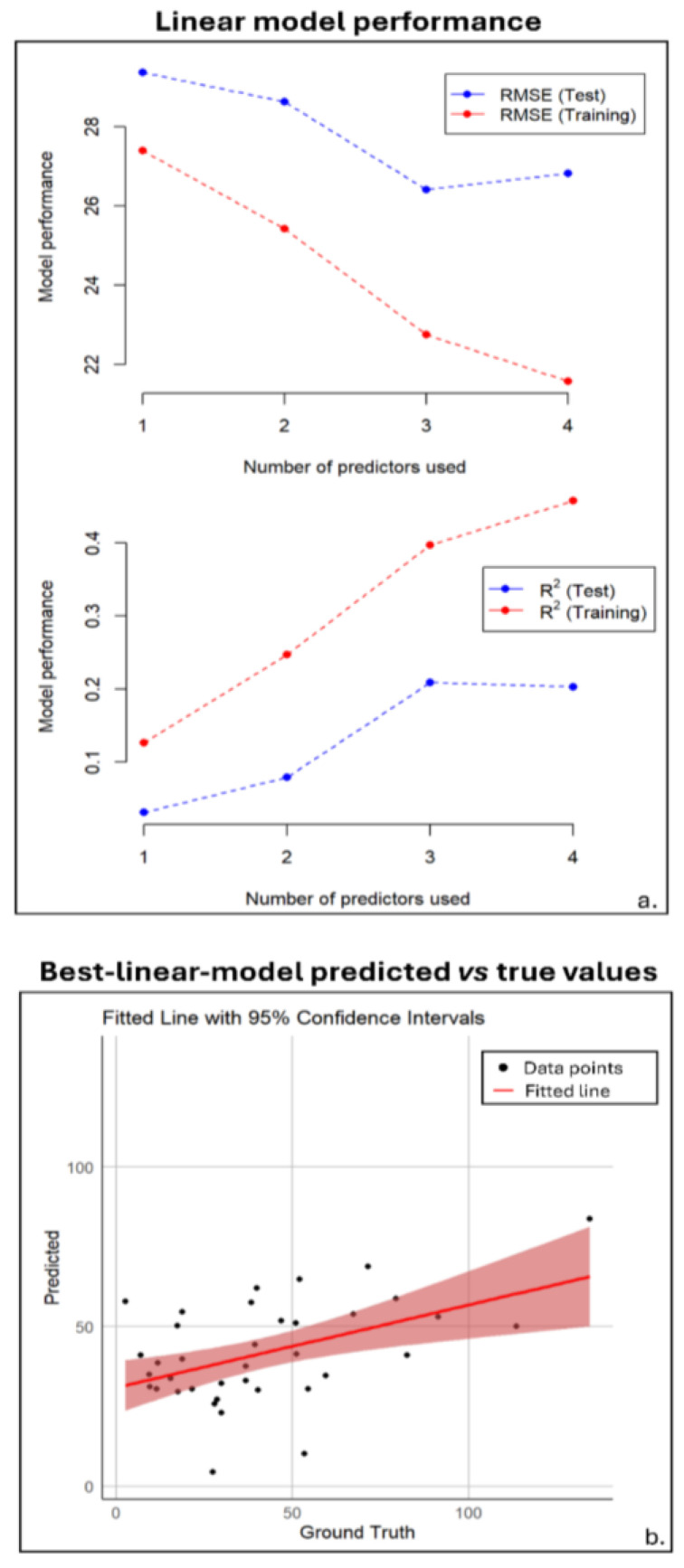
Linear model performance. Panel (**a**) displays the root-mean-squared error (RMSE) and R^2^ values for both training (red) and test (blue) sets, showing the performance based on the number of predictors. The test set values were obtained by leaving out one data point and training on the rest, while the training values are averaged across all training samples. Panel (**b**) presents a scatter plot of the predicted versus ground truth values for the best model, which uses three predictors, and includes the regression line with a 95% confidence interval.

The RMSE and R^2^ values were reported for training and validation sets across four LR models with up to four predictors in panel a. of Figure 3. Training and validation RMSEs decreased with up to three predictors (RMSE = 26.4, R^2^ = 0.21), but validation increased with four, suggesting overfitting.

**Figure 4:**
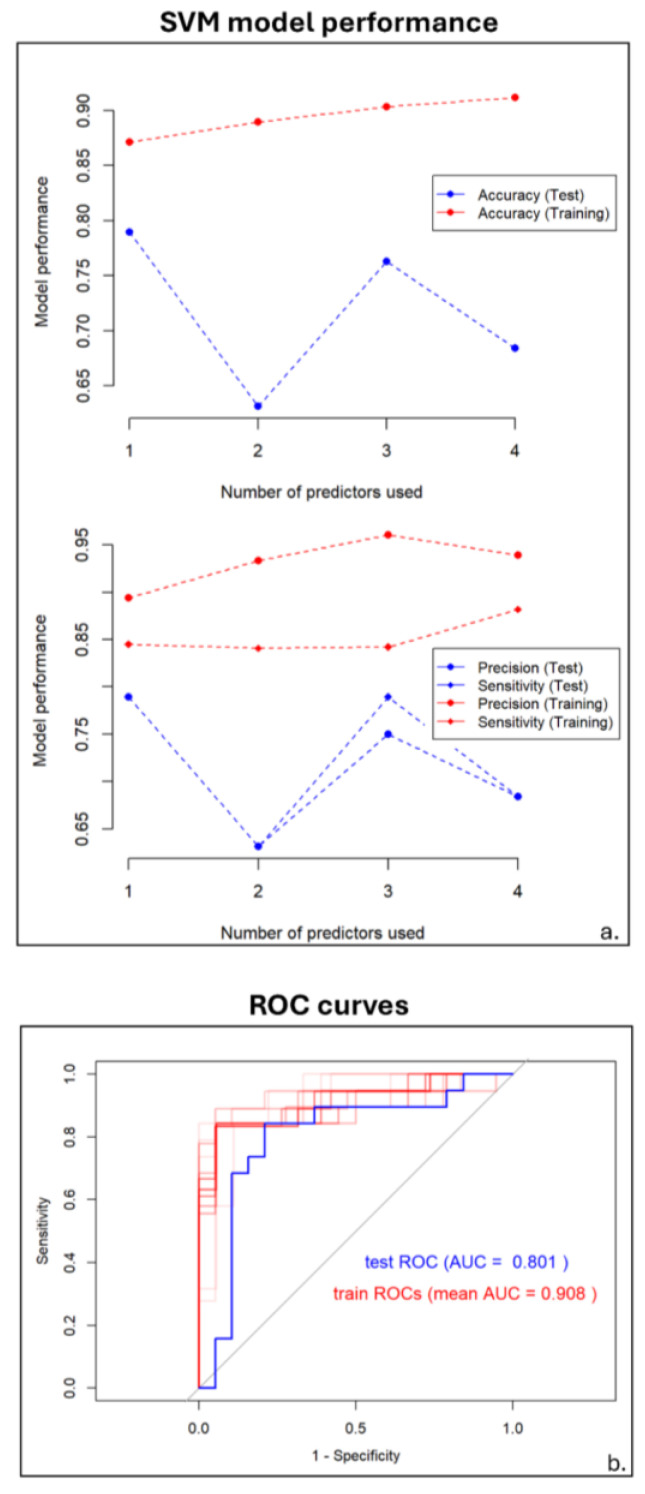
Support vector machine (SVM) model performance. Similar to Figure 1 but for SVM. In panel (**a**), performance is computed in terms of accuracy, precision and sensitivity. Panel (**b**) presents the receiver operating characteristic (ROC) curve for the test set (blue) and the ROC curves for each training sample (red, with transparency), including the area under the curves (AUC).

In relation to the results of training and validating of the SVM model, the best performance was achieved with one predictor, yielding an accuracy, precision and sensitivity of 0.79 on the test set. Higher predictor counts led to overfitting, with the best SVM model showing an AUC of 0.80 for the test set and an average AUC of 0.91 for the training sets (Figure 4).

### 3.5. Kaplan–Meier Analysis

**Figure 5:**
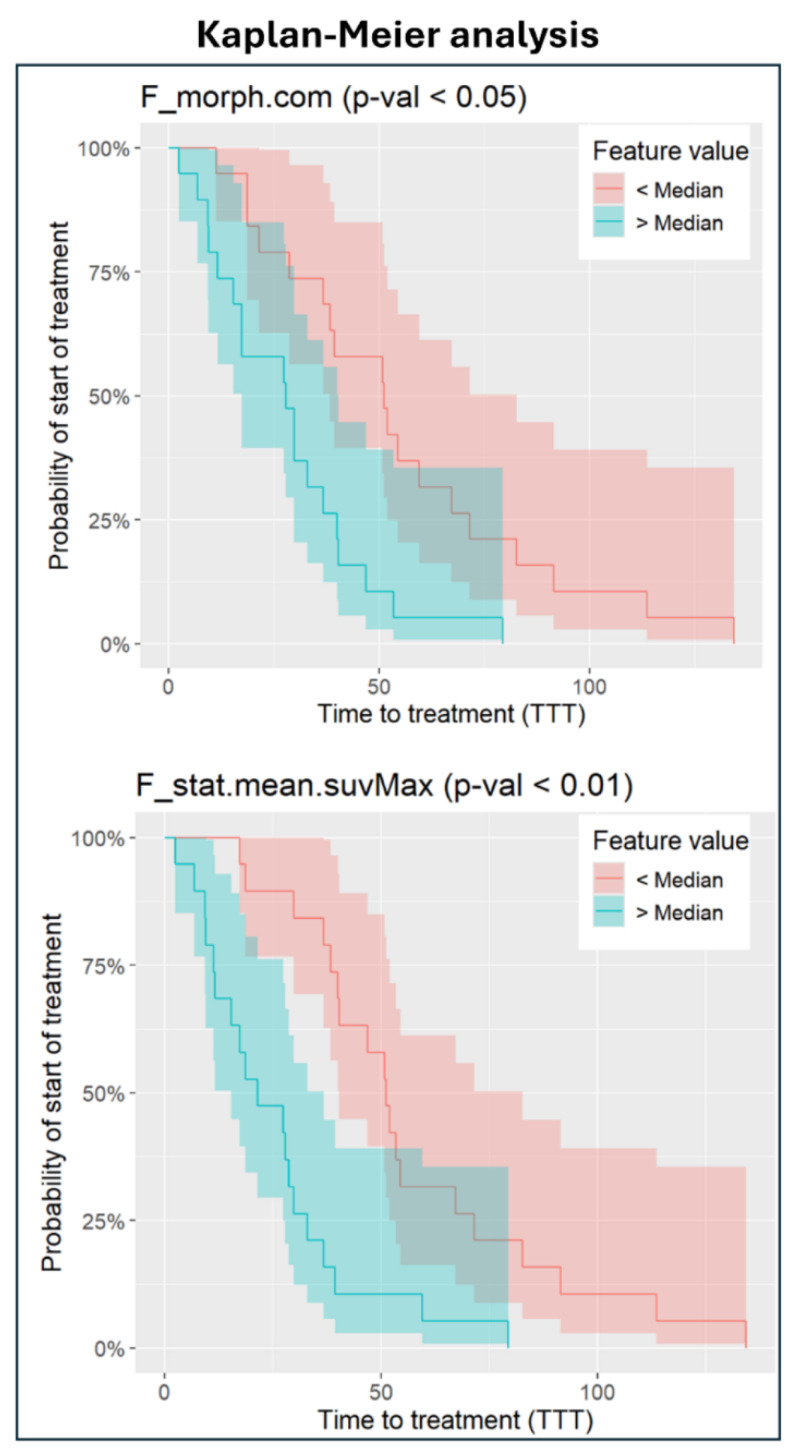
Kaplan–Meier analysis results. The top plot shows the Kaplan–Meier curve based on the centre of mass shift, a morphological feature. The bottom plot displays the Kaplan–Meier curve based on the mean PET-intensity in the volume of interest (VOI) containing the SUVmax value. A log-rank test was used to determine if there were statistically significant differences between the curves, with the null hypothesis of no difference being rejected in both cases. The *p*-values were adjusted for multiple comparisons.

Twenty-six out of thirty-eight patients started the treatment and were considered for the analysis. Patients were separated into two groups according to PET-derived radiomic features, as shown in Figure 5. In seven cases, these features significantly differentiated patient survival curves as follows: one feature was the centre of mass shift (*p* < 0.05) computed over the entire tumour burden, and six features were derived from the SUVmax VOI, including mean value (*p* < 0.02), median value (*p* < 0.02), inter-quartile range (*p* < 0.04), mean-absolute deviation (*p* < 0.04), robust-mean-absolute deviation (*p* < 0.02) and root-mean-squared (*p* < 0.02).

## 4. Discussion

This single-centre preliminary retrospective study explored the association of radiomic features extracted from the baseline metabolic tumour burden as well as from the healthy liver with TTT of FL patients in a WW strategy. Our data suggested that radiomic features from FDG PET/CT at diagnosis could be helpful to identify a subgroup of patients who will require treatment within a shorter time and demonstrate their added value to the clinical prognostic indices.

To our knowledge, this is the first study in the literature proposing to use radiomics from baseline PET/CT in low-burden FL for the prediction of starting treatment in patients on WW strategy. Previous reports investigated the role of the texture analysis in high-burden FL from CT images of baseline PET/CT scans [33], or exploited features from PET images in patients treated with first-line immuno-chemotherapy, including anti-CD20 monoclonal antibodies [12].

Noteworthy, in this study we showed that features computed from the metabolic tumour burden, from the lesion with the highest SUVmax and from the healthy liver could be combined to construct predictive models. We constructed two types of models, one designed to predict the continuous TTT value (the LR model), and another designed to predict whether the TTT could be found before or after a pre-determined TTT threshold value (the SVM model). The latter model had overall good performance, while the former model, due to its inherent greater complexity as well as to the limited number of cases available, performed slightly worse.

We analysed variants of the models employing different number of features hence different degrees of complexity. For the LR model, the best performing model (RMSE = 26.4) employed three radiomic features, one obtained from the TMTV and two from the liver. The extraction of features from the liver was motivated by previous studies supporting the so-called “theft hypothesis”, whereby FDG-avid tumour masses would deprive healthy organs such as the liver and the cerebellum of FDG, showing that the uptake of healthy organs could potentially substitute more conventional parameters as prognosis predictors [21,22]. The “theft hypothesis” could explain the presence of liver-derived features as predictors in the LR model; more specifically, the minimum found in the VOI and the skewness of the SUV distribution. To support the hypothesis, some of the features extracted from the liver were found to correlate with the features extracted from the total burden and from the SUVmax lesions (Figures S1 and S2 in the Supplementary Materials). Probably, this is one of the first approaches involving the healthy liver uptake in the radiomic analysis with a predictive role, although its use should be considered with caution as it could be affected by metabolic diseases.

The best SVM model was the one including one feature only (AUC = 0.80), extracted from the VOI that contained the maximum SUV value over the entire tumour burden. Specifically, the selected feature was the median SUV value within that lesion. Previous studies have already demonstrated the prognostic role of SUVmax in different lymphoma histotypes such as classical Hodgkin’s Lymphoma [17,18]. Interestingly, the results presented in this study suggested that radiomic parameters from the whole lesion containing the SUVmax may have a predictive value, beyond that of the SUVmax alone. Achieving this result might be relevant as FL patients are often challenging, showing several lesions of different sizes and not homogeneous radiopharmaceutical uptake. As a result, radiomics from the lesion containing the SUVmax may assume a strategic role in this scenario.

The results of the Kaplan–Meier analysis showed that radiomics might have the potential of stratifying the cohort showing different time of progressive disease. Moreover, it was confirmed that most of the significant features were extracted from the lesion with the highest SUVmax. Interestingly, a morphological feature was also found significant in this analysis; i.e., the centre of mass shift, which measures the shift between the VOI centre of the tumour burden and the SUVmax in the VOI. This result may be related to the influence of the morphological and spatial distribution of the lesions on the time to progression.

Various semi-quantitative parameters extracted from PET/CT have been proposed with a prognostic purpose to assess the risk of progression and early death related to FL. The TMTV, which represents the volume of all FDG-avid lesions, has been often considered in order to predict FL patients’ outcome from the beginning, but with conflicting results [17]. For example, no consensus has been found on the optimal TMTV cut points for TTT prediction: Meignan et al. [11] selected a TMTV threshold of 510 cm^3^ as the optimal cut-off for progression-free survival and overall survival in FL patients, with no mention regarding the extent of the tumour; So et al. [19] found that the optimal cut-off for predicting such events was 121.1 cm^3^, while two different studies reported a cut-off value of 14 cm^3^ [15,20].

As largely reported, creating radiomic models and extracting features are a complex process and require a long and rigorous procedure. Several methodological modalities have been used for the purpose, implying heterogeneous results. The variability of target volume delineation protocols and the difficulty in methodological harmonization limited the application of radiomics in clinical practice [34,35,36]. The use of different scanners can be considered a further confounding factor. Indeed, pooling images from different scanners is not simple, as some quantitative parameters (SUV, TMTV) have been demonstrated to be influenced by the scanner in use [37,38]. Different procedures have been proposed for harmonization; in this work, the ComBat harmonization method was implemented, which in the field is considered the most effective [29,31,37]. The results of the harmonization showed that the difference between scanners was reduced making the results more reliable.

Some limitations of this study should be acknowledged. The focus of this study was on a relatively uncommon type of lymphoma and on the subgroup of the untreated patients, which limited the accessibility to a large cohort. Our results were hence limited by the small sample size that represented the lower-bound to construct a reliable prediction model [39] as well as the retrospective evaluation approach [40]. Nonetheless, the implementation of the leave-one-out cross-validation approach allowed us to obtain an internal validation, despite the limited number of patients. Future studies should confirm the reliability of the models constructed in this study on a larger sample size. Another desirable component that was not included in this work was the use of an external validation that could be leveraged to assess the generalizability of the observed results to data acquired from other institutions. Further analyses should include such external dataset. Lastly, it is mandatory to explore the biologic mechanism underlying the radiomic features and their possible correlations with the clinical outcome. Multi-centre studies with larger cohorts may allow for stronger results and validate radiomics integration with the FLIPI score and other clinical parameters.

## 5. Conclusions

In this preliminary study, PET/CT radiomics obtained from the baseline metabolic tumour burden and the most active lesion as well as from the healthy liver could predict TTT of FL patients on a WW strategy. Therefore, these imaging-derived parameters might support decision-making in therapeutic strategies of patients in a similar setting.

## Figures and Tables

**Table 1:** Baseline patient and disease’ characteristics.

Age (Years), Mean ± SD * (Range)	61 ± 12 (34–85)
**Sex**, number (%) - Male - Female	19 (50)19 (50)
**Grading of the disease**, number (%) - G1 - G2 - G3 - others	4 (11)26 (68)5 (13)3 (8)
**Bone involvement**, number (%) - Yes - No - N/A †	8 (21)29 (76)1 (3)
**Stage disease**, number (%) - I - II - III - IV	3 (8)10 (26)10 (26)15 (40)
**FLIPI** ‡, number (%) - 0 - 1 - 2 - 3	3 (8)12 (32)13 (34)10 (26)

* SD: standard deviation, † N/A: not available, ‡ FLIPI: Follicular Lymphoma International Prognostic Index.

**Table 2:** Baseline PET/CT parameters.

**Contoured lesions,** n. 427	7 (2–13)
SUVmax *	10.79 (6.91–13.98)
**SUVmean** †	3.53 (3.49–5.79)
**TMTV** ‡, cm^3^	22.82 (6.16–52.03)

Data are calculated as median and interquartile range (in brackets). * SUVmax = maximum standardized uptake value; † SUVmean = mean standardized uptake value; ‡ TMTV = total metabolic tumour volume.

**Table 3:** Linear regression (LR) and support vector machine (SVM) model performance. The performance obtained for training and test sets are reported in terms of root-mean-squared error (RMSE) and R^2^ for the LR model and in terms of accuracy (acc.), precision (prec.), sensitivity (sens.) and area under the curve (AUC) for the SVM model. We detailed the performances for four variations of the models, using an increasing number of features, from one to four.

**LR Model**								
	**Training**				**Testing**			
**N. Feature**	**RMSE**		**R^2^**		**RMSE**		**R^2^**	
**1**	27.398		0.126		29.365		0.031	
**2**	25.426		0.247		28.627		0.079	
**3**	22.752		0.397		26.414		0.209	
**4**	21.580		0.458		26.817		0.202	
**SVM Model**								
	**Training**				**Testing**			
**N. feature**	**Acc.**	**Prec.**	**Sens.**	**AUC**	**Acc.**	**Prec.**	**Sens.**	**AUC**
**1**	0.870	0.894	0.845	0.908	0.789	0.789	0.789	0.801
**2**	0.890	0.933	0.841	0.910	0.632	0.632	0.632	0.723
**3**	0.903	0.960	0.842	0.926	0.763	0.750	0.789	0.765
**4**	0.912	0.939	0.882	0.964	0.684	0.684	0.684	0.626

## Data Availability

Dataset is available at reasonable request.

## Supplementary Materials

The following supporting information can be downloaded at: https://www.mdpi.com/article/10.3390/diagnostics15040432/s1. Extracted features using IBSI-compliant MODDICOM software; Association between clinical and radiomics features; Correlation between liver-derived features; Correlation between liver-derived features and the other features.

## References

[1] Ekberg S., E Smedby K., Glimelius I., Nilsson-Ehle H., Goldkuhl C., Lewerin C., Jerkeman M., Eloranta S. (2020). Trends in the prevalence, incidence and survival of non-Hodgkin lymphoma subtypes during the 21st century—A Swedish lymphoma register study. Br. J. Haematol..

[2] Freedman A. (2015). Follicular lymphoma: 2015 update on diagnosis and management. Am. J. Hematol..

[3] Link B.K., Maurer M.J., Nowakowski G.S., Ansell S.M., Macon W.R., Syrbu S.I., Slager S.L., Thompson C.A., Inwards D.J., Johnston P.B. (2013). Rates and outcomes of follicular lymphoma transformation in the immunochemotherapy era: A report from the University of Iowa/MayoClinic Specialized Program of Research Excellence Molecular Epidemiology Resource. J. Clin. Oncol..

[4] Brice P., Bastion Y., Lepage E., Brousse N., Haïoun C., Moreau P., Straetmans N., Tilly H., Tabah I., Solal-Céligny P. (1997). Comparison in low-tumor-burden follicular lymphomas between an initial no-treatment policy, prednimustine, or interferon alfa: A randomized study from the Groupe d’Etude des Lymphomes Folliculaires. Groupe d’Etude des Lymphomes de l’Adulte. J. Clin. Oncol..

[5] Solal-Céligny P., Roy P., Colombat P., White J., Armitage J.O., Arranz-Saez R., Au W.Y., Bellei M., Brice P., Caballero D. (2004). Follicular lymphoma international prognostic index. Blood.

[6] Federico M., Bellei M., Marcheselli L., Luminari S., Lopez-Guillermo A., Vitolo U., Pro B., Pileri S., Pulsoni A., Soubeyran P. (2009). Follicular lymphoma international prognostic index 2: A new prognostic index for follicular lymphoma developed by the international follicular lymphoma prognostic factor project. J. Clin. Oncol..

[7] Solal-Céligny P., Bellei M., Marcheselli L., Pesce E.A., Pileri S., McLaughlin P., Luminari S., Pro B., Montoto S., Ferreri A.J. (2012). Watchful waiting in low-tumor burden follicular lymphoma in the rituximab era: Results of an F2-study database. J. Clin. Oncol..

[8] Barrington S.F., Mikhaeel N.G., Kostakoglu L., Meignan M., Hutchings M., Müeller S.P., Schwartz L.H., Zucca E., Fisher R.I., Trotman J. (2014). Role of imaging in the staging and response assessment of lymphoma: Consensus of the International Conference on Malignant Lymphomas Imaging Working Group. J. Clin. Oncol..

[9] Cheson B.D., Fisher R.I., Barrington S.F., Cavalli F., Schwartz L.H., Zucca E., Lister T.A., Alliance, Australasian Leukaemia and Lymphoma Group, Eastern Cooperative Oncology Group, European Mantle Cell Lymphoma Consortium (2014). Recommendations for initial evaluation, staging, and response assessment of Hodgkin and non-Hodgkin lymphoma: The Lugano classification. J. Clin. Oncol..

[10] Strati P., Ahmed M.A., Fowler N.H., Nastoupil L.J., Samaniego F., Fayad L.E., Hagemeister F.B., Romaguera J.E., Rodriguez A., Wang M. (2020). Pre-treatment maximum standardized uptake value predicts outcome after frontline therapy in patients with advanced stage follicular lymphoma. Haematologica.

[11] Meignan M., Cottereau A.S., Versari A., Chartier L., Dupuis J., Boussetta S., Grassi I., Casasnovas R.O., Haioun C., Tilly H. (2016). Baseline Metabolic Tumor Volume Predicts Outcome in High-Tumor-Burden Follicular Lymphoma: A Pooled Analysis of Three Multicenter Studies. J. Clin. Oncol..

[12] Draye-Carbonnier S., Camus V., Becker S., Tonnelet D., Lévêque E., Zduniak A., Jardin F., Tilly H., Vera P., Decazes P. (2024). Prognostic value of the combination of volume, massiveness and fragmentation parameters measured on baseline FDG pet in high-burden follicular lymphoma. Sci. Rep..

[13] Liang J.H., Zhang Y.P., Xia J., Ding C.Y., Wu W., Wang L., Cao L., Zhu H.Y., Fan L., Li T.N. (2019). Prognostic Value of Baseline and Interim Total Metabolic Tumor Volume and Total Lesion Glycolysis Measured on ^18^F-FDG PET-CT in Patients with Follicular Lymphoma. Cancer Res. Treat..

[14] Parekh V., Jacobs M.A. (2016). Radiomics: A new application from established techniques. Expert Rev. Precis. Med. Drug Dev..

[15] Leccisotti L., Maccora D., Malafronte R., D’Alò F., Maiolo E., Annunziata S., Rufini V., Giordano A., Hohaus S. (2023). Predicting time to treatment in follicular lymphoma on watchful waiting using baseline metabolic tumour burden. J. Cancer Res. Clin. Oncol..

[16] Major A., Hammes A., Schmidt M.Q., Morgan R., Abbott D., Kamdar M. (2020). Evaluating Novel PET-CT Functional Parameters TLG and TMTV in Differentiating Low-grade Versus Grade 3A Follicular Lymphoma. Clin. Lymphoma Myeloma Leuk..

[17] Prieto Prieto J.C., Vallejo Casas J.A., Hatzimichael E., Fotopoulos A., Kiortsis D.N., Sioka C. (2020). The contribution of metabolic parameters of FDG PET/CT prior and during therapy of adult patients with lymphomas. Ann. Nucl. Med..

[18] Mettler J., Müller H., Voltin C.A., Baues C., Klaeser B., Moccia A., Borchmann P., Engert A., Kuhnert G., Drzezga A.E. (2018). Metabolic Tumour Volume for Response Prediction in Advanced-Stage Hodgkin Lymphoma. J. Nucl. Med..

[19] So H., Lee H., Hyun S.H., Cho Y.S., Moon S.H., Choi J.Y., Lee K.H. (2023). Metabolic bulk volume from FDG PET as an independent predictor of progression-free survival in follicular lymphoma. Front. Oncol..

[20] Rodier C., Kanagaratnam L., Morland D., Herbin A., Durand A., Chauchet A., Choquet S., Colin P., Casasnovas R.O., Deconinck E. (2023). Risk Factors of Progression in Low-tumor Burden Follicular Lymphoma Initially Managed by Watch and Wait in the Era of PET and Rituximab. Hemasphere.

[21] Morland D., Triumbari E.K.A., Maiolo E., Cuccaro A., Treglia G., Hohaus S., Annunziata S. (2022). Healthy Organs Uptake on Baseline ^18^F-FDG PET/CT as an Alternative to Total Metabolic Tumor Volume to Predict Event-Free Survival in Classical Hodgkin’s Lymphoma. Front. Med..

[22] Godard F., Durot E., Durot C., Hoeffel C., Delmer A., Morland D. (2022). Cerebellum/liver index in pretherapeutic ^18^F-FDG PET/CT as a predictive marker of progression-free survival in follicular lymphoma treated by immunochemotherapy and rituximab maintenance. Medicine.

[23] Boellaard R., Delgado-Bolton R., Oyen W.J., Giammarile F., Tatsch K., Eschner W., Verzijlbergen F.J., Barrington S.F., Pike L.C., Weber W.A. (2015). FDG PET/CT: EANM procedure guidelines for tumour imaging: Version 2.0. Eur. J. Nucl. Med. Mol. Imaging.

[24] Werner-Wasik M., Nelson A.D., Choi W., Arai Y., Faulhaber P.F., Kang P., Almeida F.D., Xiao Y., Ohri N., Brockway K.D. (2012). What is the best way to contour lung tumors on PET scans? Multiobserver validation of a gradient-based method using a NSCLC digital PET phantom. Int. J. Radiat. Oncol. Biol. Phys..

[25] Zwanenburg A., Vallières M., Abdalah M.A., Aerts H.J.W.L., Andrearczyk V., Apte A., Ashrafinia S., Bakas S., Beukinga R.J., Boellaard R. (2020). The Image Biomarker Standardization Initiative: Standardized Quantitative Radiomics for High-Throughput Image-based Phenotyping. Radiology.

[26] Dinapoli N., Alitto A.R., Vallati M., Gatta R., Autorino R., Boldrini L., Damiani A., Valentini V. (2015). Moddicom: A complete and easily accessible library for prognostic evaluations relying on image features. Annu. Int. Conf. IEEE Eng. Med. Biol. Soc..

[27] Johnson W.E., Li C., Rabinovic A. (2007). Adjusting batch effects in microarray expression data using empirical Bayes methods. Biostatistics.

[28] Fortin J.P., Cullen N., Sheline Y.I., Taylor W.D., Aselcioglu I., Cook P.A., Adams P., Cooper C., Fava M., McGrath P.J. (2018). Harmonization of cortical thickness measurements across scanners and sites. Neuroimage.

[29] Orlhac F., Boughdad S., Philippe C., Stalla-Bourdillon H., Nioche C., Champion L., Soussan M., Frouin F., Frouin V., Buvat I. (2018). A Postreconstruction Harmonization Method for Multicenter Radiomic Studies in PET. J. Nucl. Med..

[30] Orlhac F., Frouin F., Nioche C., Ayache N., Buvat I. (2019). Validation of A Method to Compensate Multicenter Effects Affecting CT Radiomics. Radiology.

[31] Orlhac F., Lecler A., Savatovski J., Goya-Outi J., Nioche C., Charbonneau F., Ayache N., Frouin F., Duron L., Buvat I. (2021). How can we combat multicenter variability in MR radiomics? Validation of a correction procedure. Eur. Radiol..

[32] Mahon R.N., Ghita M., Hugo G.D., Weiss E. (2020). ComBat harmonization for radiomic features in independent phantom and lung cancer patient computed tomography datasets. Phys. Med. Biol..

[33] Durot C., Durot E., Mulé S., Morland D., Godard F., Quinquenel A., Delmer A., Soyer P., Hoeffel C. (2023). Pretreatment CT Texture Parameters as Predictive Biomarkers of Progression-Free Survival in Follicular Lymphoma Treated with Immunochemotherapy and Rituximab Maintenance. Diagnostics.

[34] Zwanenburg A. (2019). Radiomics in nuclear medicine: Robustness, reproducibility, standardization, and how to avoid data analysis traps and replication crisis. Eur. J. Nucl. Med. Mol. Imaging.

[35] Triumbari E.K.A., Gatta R., Maiolo E., De Summa M., Boldrini L., Mayerhoefer M.E., Hohaus S., Nardo L., Morland D., Annunziata S. (2023). Baseline ^18^F-FDG PET/CT Radiomics in Classical Hodgkin’s Lymphoma: The Predictive Role of the Largest and the Hottest Lesions. Diagnostics.

[36] Rizzo A., Triumbari E.K.A., Gatta R., Boldrini L., Racca M., Mayerhoefer M., Annunziata S. (2021). The role of ^18^F-FDG PET/CT radiomics in lymphoma. Clin. Transl. Imaging.

[37] Orlhac F., Eertink J.J., Cottereau A.S., Zijlstra J.M., Thieblemont C., Meignan M., Boellaard R., Buvat I. (2022). A Guide to ComBat Harmonization of Imaging Biomarkers in Multicenter Studies. J. Nucl. Med..

[38] Shiri I., Rahmim A., Ghaffarian P., Geramifar P., Abdollahi H., Bitarafan-Rajabi A. (2017). The impact of image reconstruction settings on ^18^F-FDG PET radiomic features: Multi-scanner phantom and patient studies. Eur. Radiol..

[39] An C., Park Y.W., Ahn S.S., Han K., Kim H., Lee S.K. (2021). Radiomics machine learning study with a small sample size: Single random training-test set split may lead to unreliable results. PLoS ONE.

[40] Ward R.A., Brier M.E. (1999). Retrospective analyses of large medical databases: What do they tell us?. J. Am. Soc. Nephrol..

