# Supplementary material for: The Predictive Role of Baseline 18F-FDG PET/CT Radiomics in Follicular Lymphoma on Watchful Waiting: A Preliminary Study
Source: Diagnostics (Basel). 2025 Feb 11;15(4):432. doi: 10.3390/diagnostics15040432 (PMC11854662; doi:10.3390/diagnostics15040432)
Supplement: Supplementary file 1 [file diagnostics-15-00432-s001.zip › diagnostics-3330963-supplementary.pdf]

Extracted features using IBSI-compliant MODDICOM software

- Intensity-based statistical features

- Mean
- Variance
- Skewness
- Kurtosis
- Median
- Minimum grey level
- 10th percentile
- 90th percentile
- Maximum grey level
- Interquartile range
- Range
- Mean absolute deviation
- Robust mean absolute deviation
- Energy
- Root mean square
- Entropy
- Uniformity

- Morphological features

- Volume
- Surface area
- Surface to volume ratio
- Compactness 1
- Compactness 2
- Spherical disproportion
- Sphericity
- Asphericity
- Centre of mass shift
- Maximum 3D diameter
- Major axis length
- Minor axis length
- Least axis length
- Elongation
- Flatness

- Texture features - Grey level co-occurrence based features

- Joint maximum
- Joint average

- Joint variance
- Joint entropy
- Difference average
- Difference variance
- Difference entropy
- Sum average
- Sum variance
- Sum entropy
- Angular second moment
- Contrast
- Dissimilarity
- Inverse difference
- Inverse difference normalised
- Inverse difference moment
- Normalised inverse difference moment
- Inverse variance
- Correlation
- Autocorrelation
- Cluster tendency
- Cluster shade
- Cluster prominence
- First measure of information correlation
- Second measure of information correlation

• Texture features - Grey level run length based features

- Short runs emphasis
- Long runs emphasis
- Low grey level run emphasis
- High grey level run emphasis
- Short run low grey level emphasis
- Short run high grey level emphasis
- Long run low grey level emphasis
- Long run high grey level emphasis
- Grey level non-uniformity
- Normalised grey level non-uniformity
- Grey level non-uniformity normalised
- Run length non-uniformity
- Run length non-uniformity normalised
- Run percentage
- Grey level variance

- Run length variance
- Run entropy

- Texture features - Grey level size zone based features

- Small zone emphasis
- Large zone emphasis
- Low grey level zone emphasis
- High grey level zone emphasis
- Small zone low grey level emphasis
- Small zone high grey level emphasis
- Large zone low grey level emphasis
- Large zone high grey level emphasis
- Grey level non-uniformity
- Grey level non-uniformity normalised
- Zone size non-uniformity
- Zone size non-uniformity normalised
- Zone percentage
- Grey level variance
- Zone size variance
- Zone size entropy

Association between clinical and radiomics features

We assessed whether there was an association between some of the clinical variables and the radiomic features extracted from the PET images. The considered clinical variables were age, sex (male/female), haemoglobin (<12 g/dl), serum lactate dehydrogenase (LDH), follicular grading, disease bulk, bone marrow involvement, stage of disease, extensive nodal disease (4 or more lymph nodes involved), and FLIPI ≥ 2. We performed a linear correlation between the age of the patients and the feature values, testing whether the correlation coefficient was different from zero. The remaining considered clinical variables were binarized and used to divide the radiomic features in two groups. Subsequently, a Wilcoxon-Mann-Whitney test was performed to assess whether there was a significant difference between the groups. None of the clinical and pathological data, was correlated with any radiomic features.

Correlation between liver-derived features

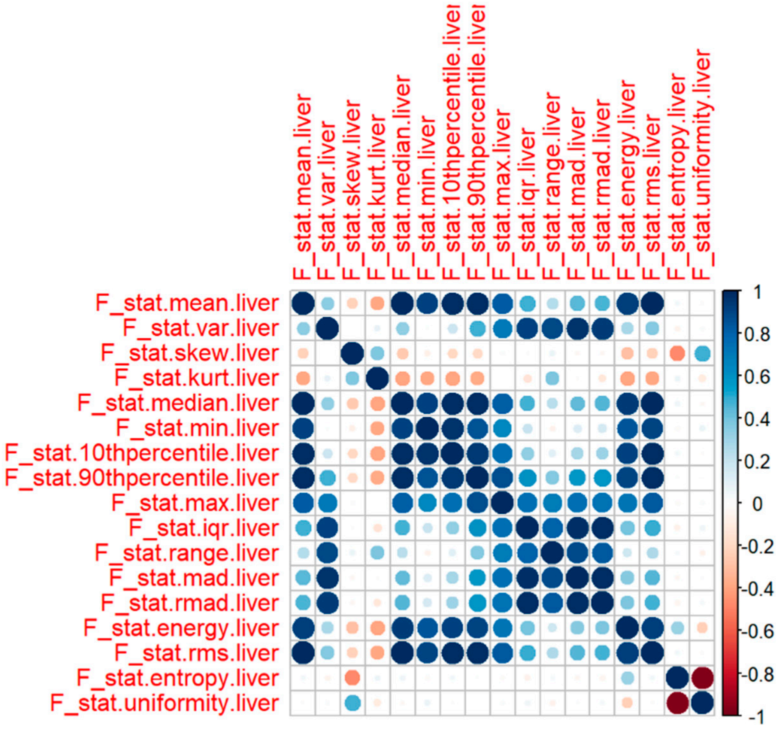

Figure S1. Levels of correlation found between the radiomic features extracted from the healthy liver volume of interest. The size and colour of the circles indicate the intensity of correlation. Blue colour represents positive correlation while red colour represents a negative correlation. Many of the features computed in the liver were found to have a strong association.

Correlation between liver-derived features and the other features

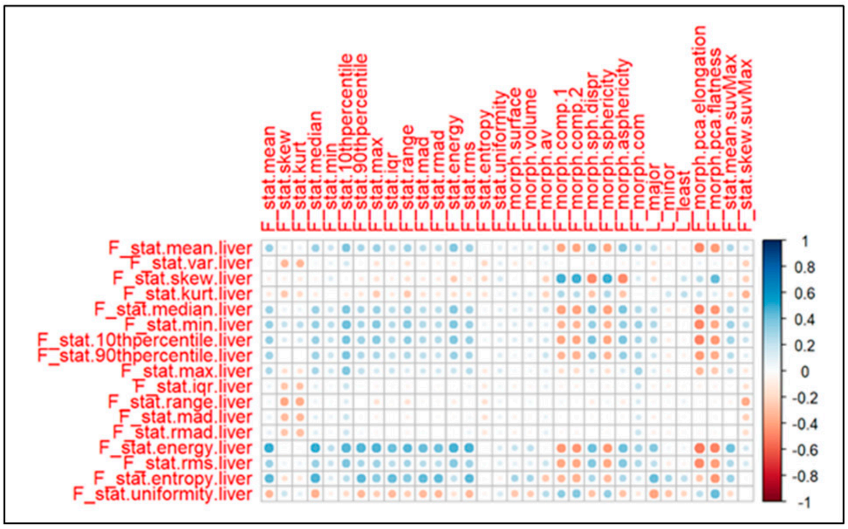

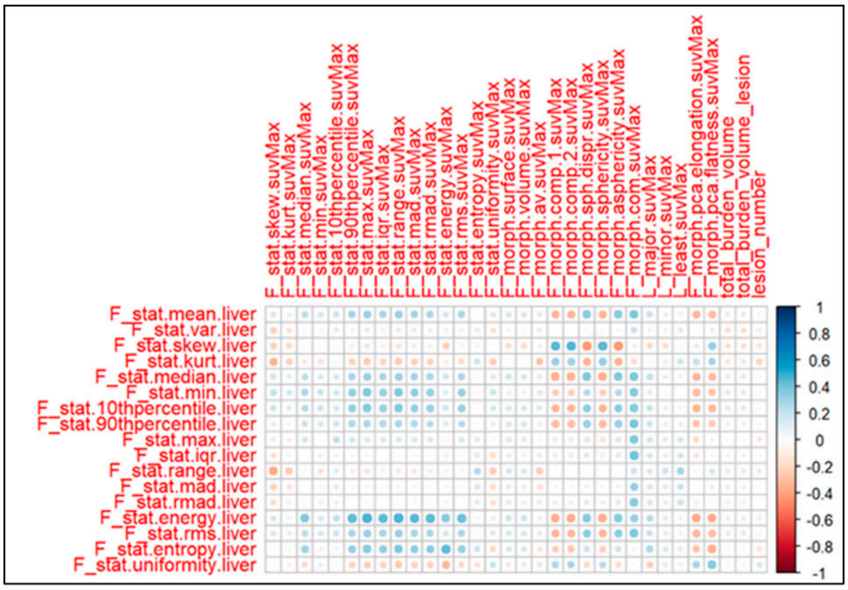

Figure S2. Correlation coefficients computed between the features obtained from the healthy liver (vertical axis) and the remaining features (horizontal axis). The non-liver features were divided into two groups and shown in different plots (top and bottom) for readability. The colour coding is similar to figure S1. Some of the features extracted from the liver were found to have a weak yet non-null correlation with the other features. Specifically, some statistical features extracted from both the total metabolic tumour burden and the lesion with highest SUVmax, as well as morphological features seem to have a stronger association.
